# Galactosialidosis: A Report of Three Cases Diagnosed With a Founder Genetic Mutation in the Bahraini Population

**DOI:** 10.7759/cureus.77750

**Published:** 2025-01-20

**Authors:** Zahra Alsahlawi, Zahraa J Alhadi, Eman A Abdulla, Sara H Ebrahim, Manal M Alshehab, Walaa R Sanad

**Affiliations:** 1 Department of Pediatrics, Arabian Gulf University, Manama, BHR; 2 Department of Pediatrics, Salmaniya Medical Complex, Manama, BHR

**Keywords:** bahraini, founder mutation, galactosialidosis, lysosomal lipid storage disease, novel mutation

## Abstract

Galactosialidosis (GS, OMIM #256540) is a rare metabolic disorder resulting from mutations in the protective protein/cathepsin A (PPCA) or CTSA gene, which is characterized by malfunction of the lysosomal glycoprotein degradation and subsequent intra-lysosomal accumulation of sialyloligosaccharides and glycopeptides. It follows an autosomal recessive inheritance pattern. This systemic disease is characterized by typical clinical features such as short stature, coarse facial features, vertebral deformities, gastrointestinal manifestations, particularly hepatosplenomegaly, cardiac abnormalities, hearing loss, and macular cherry-red spots.

GS is classified into three subtypes based on the age of onset and presenting symptoms. The three types include the early infantile (EI) form, which is the most severe; the late infantile form; and the juvenile/adult form. Here, we present three newly diagnosed cases of late-infantile GS in Bahraini patients, all sharing the same previously reported homozygous mutation in the CTSA gene (c.607C>A, p.Pro203Thr), confirmed by targeted mutation analysis. This mutation has been identified in nine Bahraini patients, reflecting a founder effect in the Bahraini population. All three patients presented with coarse facial features, short stature, and poor vision, alongside skeletal deformities. Patient 1 had significant bilateral hip osteoarthritis, while Patient 2. showed lumbar lordosis and extensive bilateral hip avascular necrosis. Patient 3 presented with thoracolumbar levoscoliosis and kyphoscoliosis. Additionally, in Patient 1 and Patient 2 cardiac manifestations were noted, including valvular heart disease. Patient 3 had mild left ventricular hypertrophy (LVH), aortic regurgitation, and mitral regurgitation, along with diffuse angiokeratomas. All patients are currently receiving supportive care and management. This case report highlights the importance of early diagnosis and multidisciplinary care of patients with GS.

## Introduction

Galactosialidosis (GS, OMIM #256540) is a rare autosomal recessive glycoprotein storage disease caused by mutations in the cathepsin A (CTSA) gene, which is composed of 15 exons [1,2]. It encodes lysosomal protective proteins [1,2]. Protective protein/cathepsin A (PPCA) forms a complex that includes β-galactosidase (β-GAL) and neuraminidase 1 (NEU1). Together, β-GAL and NEU1 facilitate the efficiency and availability of the protective cathepsin functions [1,2].

GS was first classified as a variant of GM1-gangliosidosis, a glycosphingolipid storage disease, due to an isolated deficiency of the lysosomal β-GAL [3]. However, the identification by Wenger et al. of the absence of NEU1 activity, in combination with the partial deficiency of β-GAL in a patient previously classified as having GM1-gangliosidosis, established GS as a new clinical entity, distinct from GM1-gangliosidosis [4].

GS can affect multiple organ systems, with gastrointestinal and musculoskeletal systems being the most affected [5]. Short stature, coarse facial features, skeletal deformities, and different degrees of hepatosplenomegaly followed by cardiac manifestations reported in some cases are among the initial presentations of the disease [5].

GS is classified into three subtypes based on the age of onset and clinical symptoms including early infantile (EI), late infantile, and juvenile/adult forms [5]. The EI form is characterized by early onset either in utero or within the first three months of life with progressive and rapid deterioration leading to death shortly after birth or even in utero [6-8]. In the EI form, patients may present with significant edema and abdominal swelling due to fluid accumulation (hydrops fetalis) [2,7,8]. Additional symptoms can include characteristic coarse facial features, cherry-red spots, corneal clouding, skeletal dysplasia, hepatosplenomegaly, and potential kidney or cardiac failure, which may result in death in some cases [2,6-10]. Telangiectasias are predominantly observed in this subtype [8].

In contrast, the late infantile form manifests within the first year of life, with symptoms that are generally slowly progressive into adulthood [6,8,11]. Characteristic symptoms of this subtype, alongside short stature and coarse facial features, are dysostosis multiplex, hepatosplenomegaly, cardiac involvement, hearing loss, and decreased visual acuity [2,6,8,11]. Cherry-red spots and corneal clouding may also be present, though neurological manifestations such as seizures, myoclonus, and ataxia are very rare [2,6,8].

The juvenile/adult form is the most common type of GS, predominantly observed in individuals of Japanese descent, and it varies widely in severity of symptoms and age of onset with a main presentation age in adolescence [2,6,8]. Individuals with this form typically have a normal life expectancy [2,6,8]. Besides characteristic features such as coarse facies, vertebral changes, cherry-red spots, and corneal clouding, neurological manifestations, including myoclonus, cerebellar ataxia, generalized seizures, and mental retardation, are frequently observed [2,6,8,12,13]. Potential vision and hearing loss are also common [8]. Angiokeratoma was seen primarily in this group of patients, while gastrointestinal involvement is unusual [2,6,8]. 

GS is considered one of the rarest genetic diseases with an unknown precise prevalence; however, up until the present time, nearly 157 cases have been reported worldwide, and among that number nine cases were reported from the Kingdom of Bahrain [5,8,14,15]. Here, we report three cases from two different Bahraini families: two siblings (a male and a female) and another male patient. All three patients have recently been diagnosed with GS. They all share the same genetic mutation, which has been reported in 2022 as a founder mutation in nine Bahraini patients [5]. 

## Case presentation

Case 1

A 20-year-old Bahraini male was born at full term through normal vaginal delivery with no antenatal or postnatal complications. The parents are second cousins. He was the fourth child out of five siblings (three boys and two girls). 

Birth measurements were normal. He was exclusively breastfed, and the clinical examination at birth was unremarkable. He had a history of bronchial asthma, with multiple hospital admissions for exacerbations during childhood, which resolved by the age of 11 years. 

At the age of four years, he presented to the pediatric outpatient clinic with short stature. Clinical examination showed coarse facial features and hepatomegaly, necessitating a multidisciplinary workup for suspected metabolic disease. Accordingly, an abdominal and pelvic ultrasound was conducted, showing mild hepatomegaly, a right kidney smaller than the left by about 1.5 cm, with no other significant abnormalities. Furthermore, radiographs of the skull, chest, abdomen, spine, and extremities were obtained, showing reduced bone density, short and widened clavicles, and wedge-shaped dorsolumbar vertebrae. Mild coxa valga and slightly short, widened metacarpal/metatarsal bones were noted. These findings are suggestive of dysostosis multiplex. Echocardiography at that time showed thickening of the mitral and aortic valves but without stenosis. Two years later, follow-up echocardiography revealed slight mitral stenosis and regurgitation, aortic stenosis, and tricuspid valve stenosis. Electrophoretic differentiation of mucopolysaccharides and screening for oligosaccharides in urine were performed at the ages of four and seven years, both showing normal results. Further follow-ups were lost after the age of seven.

**Figure 1:**
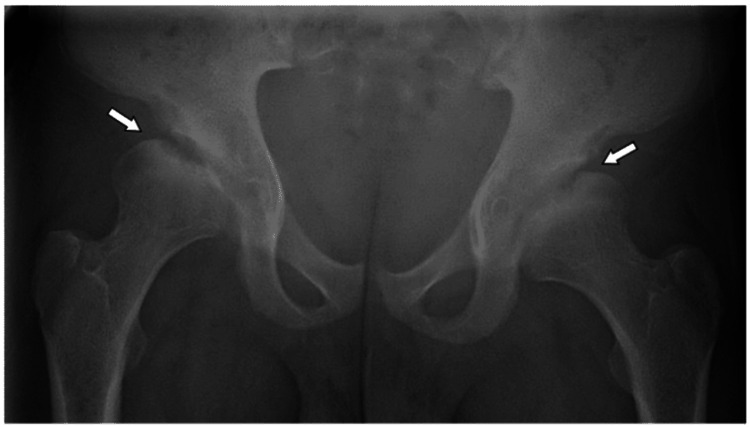
Plain pelvic X-ray of Patient 1 Bilateral avascular necrosis of the hips, demonstrating flattening of the outer portion of both femoral heads, with more significant involvement observed in the right hip.

At the age of 18, the patient experienced numbness and pain in both hands, which was diagnosed as carpal tunnel syndrome at a private hospital, resulting in a release procedure for the left hand, with the right-hand procedure pending. At the age of 19, he presented with lower back and joint pain, particularly hip pain. Multiple skeletal abnormalities and severe bilateral hip osteoarthritis were reported. Accordingly, pelvis X-rays revealed advanced bilateral hip avascular necrosis (Figure 1).

Given the clinical manifestations mentioned above, the patient was evaluated in the metabolic clinic, and GS was diagnosed clinically based on the previous experience of a founder mutation in Bahrain. Genetic testing was conducted at Bioscientia Laboratory in Germany to rule out GS at the age of 19, confirming the diagnosis through targeted mutation analysis of the CTSA gene using polymerase chain reaction. It identified a homozygous mutation (c.607C>A, p.Pro203Thr) that had been previously reported in Bahraini patients.

Currently, the patient is in generally good health but complains of back pain, which increases with long-distance walking. Genetic counseling revealed a positive family history of the same manifestations in his elder sister, which will be presented in case 2. 

Case 2

**Figure 2:**
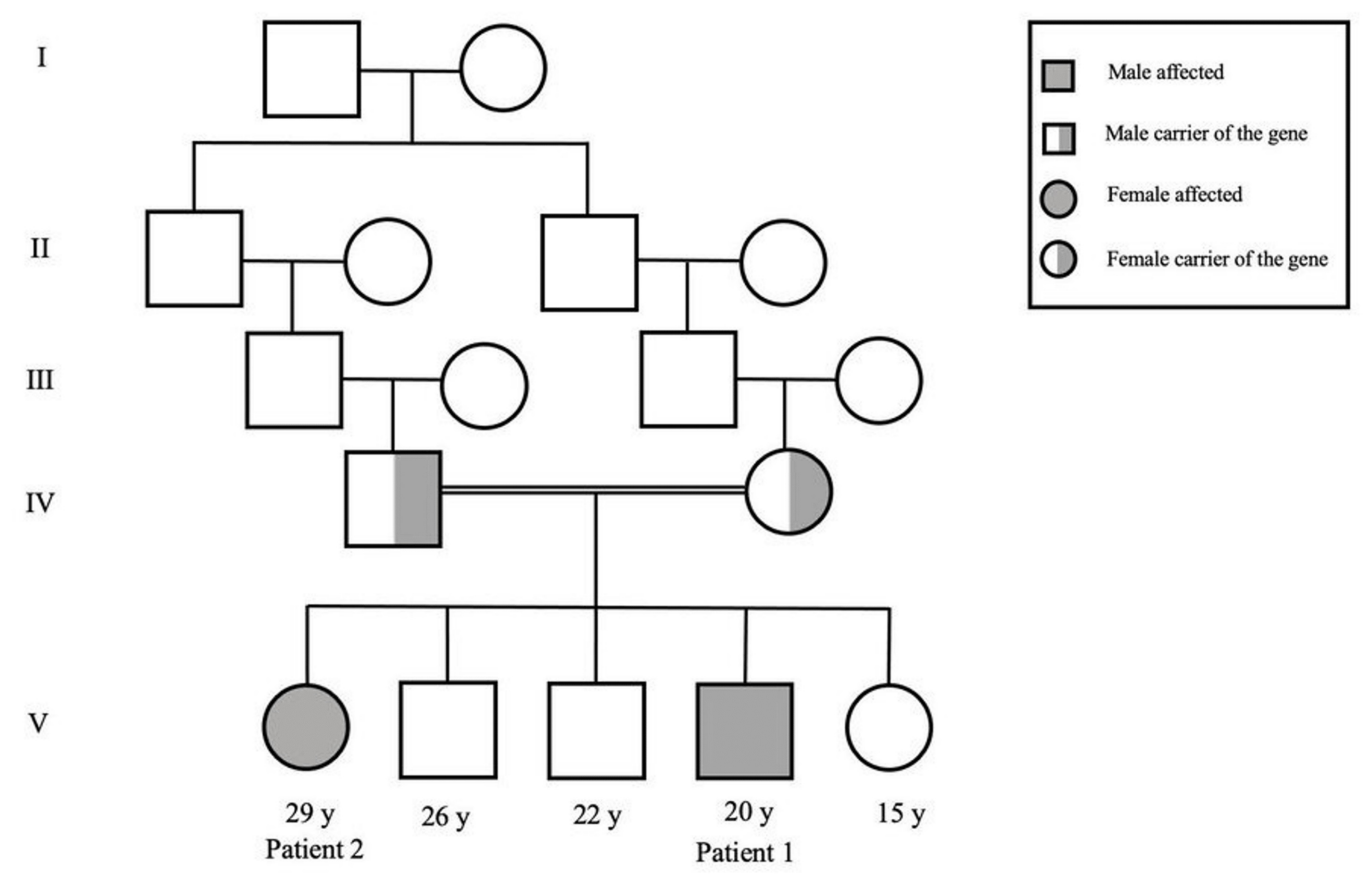
Family pedigree of two siblings with GS Image credits: Zahra Alsahlawi, Zahraa J Alhadi. GS: galactosialidosis

A 29-year-old Bahraini female, who is an older sister of Patient 1, was evaluated in the metabolic/genetic with the same clinical manifestations as her brother (Figure 2). She was born as the first child at term through normal vaginal delivery, with a birth weight of approximately 3.5 kg and no antenatal or postnatal complications, to parents who are second cousins. She was exclusively breastfed. At birth, her physical examination was unremarkable, with no abnormalities or dysmorphic features. 

She has been a known case of bronchial asthma since the age of one, for which she has been on regular follow-ups and medication. During this time, developmental delays in gross motor skills and speech were observed. She had multiple hospital admissions due to exacerbations of bronchial asthma. During her visits to the Pediatrics outpatient clinic at Salmaniya Medical Complex, laboratory investigations revealed iron deficiency anemia, which was treated with iron supplements. Furthermore, diminished hearing was also noted, and subsequent hearing assessments (tympanometry and audiometry) revealed bilateral conductive hearing loss due to excessive ear wax, which has been managed regularly since childhood.

At the age of two, during her visit to the pediatric outpatient clinic, a physical examination revealed hepatomegaly (2.5 cm below the costal margin) and coarse facial features, raising suspicion of a lysosomal storage disease, specifically mucopolysaccharidosis. A skeletal survey at that age was normal. An ejection systolic murmur (grade 2/6) was also noted, necessitating a cardiology review, which revealed pulmonary regurgitation (11 mmHg, pulmonary pressure gradient 7 mmHg), thickened mitral valve, mitral valve regurgitation (9 mmHg, mitral valve maximum gradient 18 mmHg), and tricuspid regurgitation (22 mmHg).

At the age of ten, during further follow-up, the patient presented with an abnormal gait, limping, and progressive hip and knee pain. Examinations showed short stature, mild coarse facial features, a broad nasal bridge, puffy lips, pectus carinatum, and lumbar lordosis. Furthermore, hepatosplenomegaly and ejection systolic murmur did not change. These clinical findings raised a strong suspicion of mucopolysaccharidosis. Additional work-up across multiple specialties was conducted.

At the age of 12, serial imaging studies were performed, including pelvic and knee X-rays, which revealed changes suggestive of early Perthes disease, especially in the left femoral head. Chest X-ray was normal, and lumbosacral spine imaging was suggestive of spondyloepiphyseal dysplasia congenita. A bone mineral density scan of the hands showed normal texture and density for her age.

Two years later, a magnetic resonance imaging (MRI) of the pelvis revealed bilateral significant flattening of the femoral capital epiphysis, which was symmetrical with dysplastic features of the hip. While these findings simulated Perthes disease, bone dysplasia and the possibility of spondyloepiphyseal dysplasia with mucopolysaccharidosis were considered more likely.

Laboratory investigations, including hormonal assays, were normal. A screening test for urinary glycosaminoglycans (GAGs) was sent to Frankfurt, Germany, with results showing a normal GAG pattern, thus excluding all types of mucopolysaccharidosis except MPS type IV (Morquio syndrome), which could not definitely be excluded based on clinical and radiological findings.

**Figure 3:**
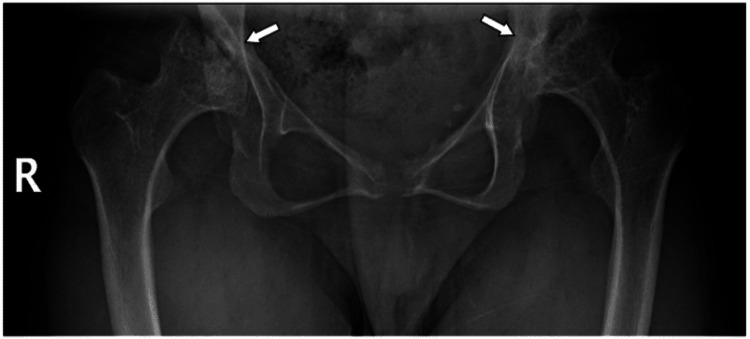
Pelvic X-ray of Patient 2 Bilateral avascular necrosis of the hips.

**Figure 4:**
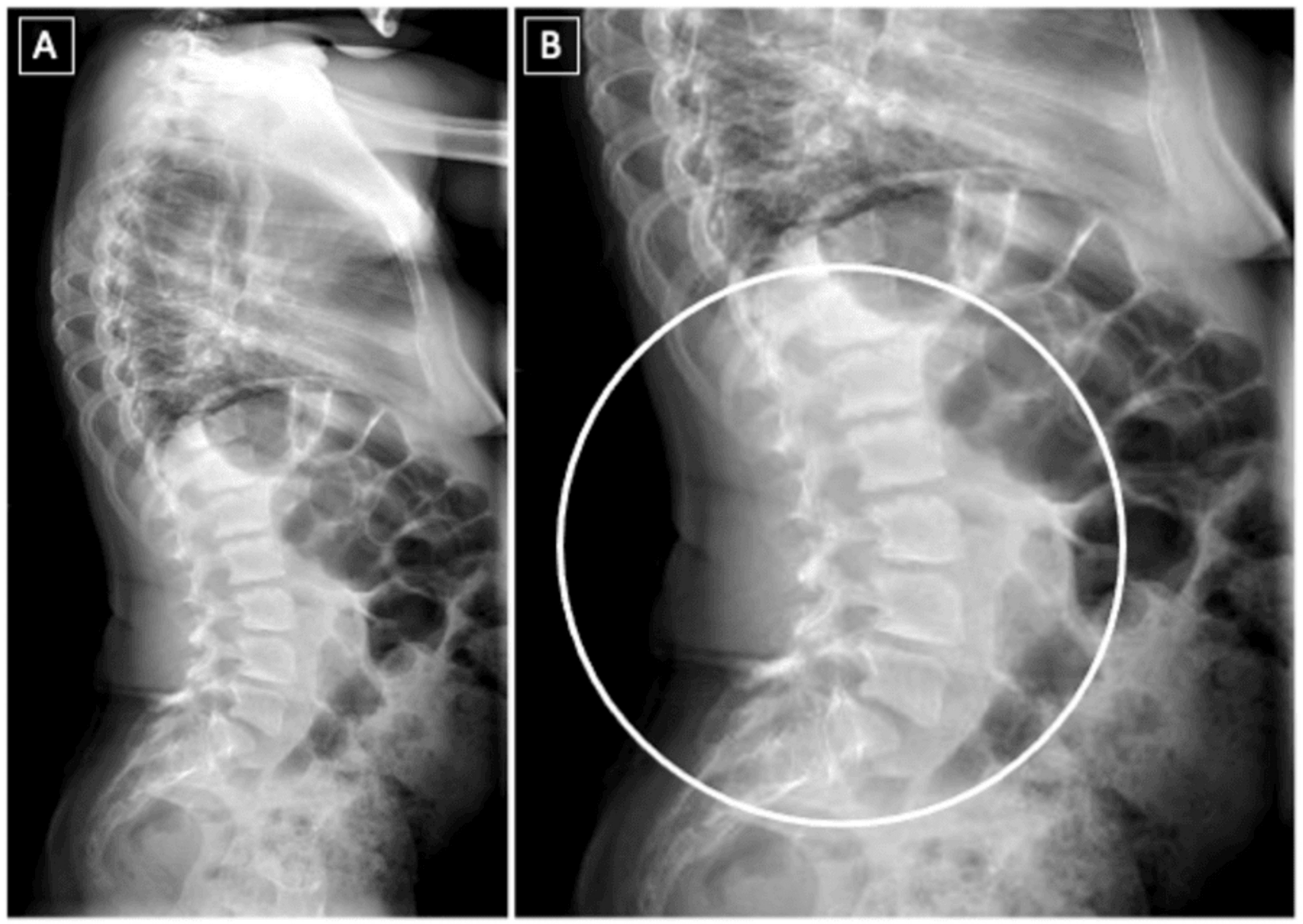
Lateral X-ray view of the whole spine of Patient 2 Lumbar lordosis, characterized by an increased sacral base angle and forward translation of the thorax.

Further investigations, including ophthalmology and cardiology reviews, were suggested but not completed, as the patient lost follow-up after the age of 15 years. Additionally, scanogram images of the lower extremities and pelvic X-rays revealed advanced bilateral hip avascular necrosis, indicating the need for total hip replacement (Figure 3). A scanogram of the full spine showed lumbar lordosis (Figure 4).

Based on the clinical presentation, laboratory results, skeletal findings, and a positive mutation in her younger brother for GS, the patient was called to attend the metabolic and genetic clinic. At the age of 28 years, coinciding with her brother's diagnosis of GS in the same year, the diagnosis of GS for her was confirmed through targeted mutation analysis using polymerase chain reaction. This genetic testing identified a homozygous mutation in the CTSAgene (c.607C>A, p.Pro203Thr).

The patient is currently stable, with no significant complications other than skeletal deformities and difficulty walking long distances, accompanied by back pain. Genetic counseling was given to the family about the risk of recurrence, other siblings have not been tested for the familial variant as they clearly do not have any symptoms related to the disease.

Case 3

An 11-year-old Bahraini male was born at the term of an uneventful second pregnancy via normal vaginal delivery with no antenatal or postnatal complications and no neonatal intensive care admission. Birth measurements were normal. The parents are first cousins, and the family history is unremarkable. He has a healthy older brother. Regarding his surgical history, the patient underwent surgery for the trigger finger on his right thumb at the age of seven in another hospital.

At the age of six, the patient presented to the Orthopedics clinic for skeletal deformities. Clinical examination showed thoracolumbar scoliosis and dysmorphic facial features, prompting further evaluation. Regular follow-ups with Orthopedics commenced and genetic consultation for skeletal deformity was initiated for the possibility of skeletal dysplasia. With the presence of dysmorphic features, genetic testing was performed by ordering a dysmorphology genes panel in Centene, which was reported as negative. 

An MRI of the whole spine performed at the age of eight confirmed scoliotic deformity with convexity to the left, while an MRI of the brain was unremarkable. Thus, the patient was diagnosed with congenital thoracolumbar kyphoscoliosis, necessitating surgery at the age of 12.

**Figure 5:**
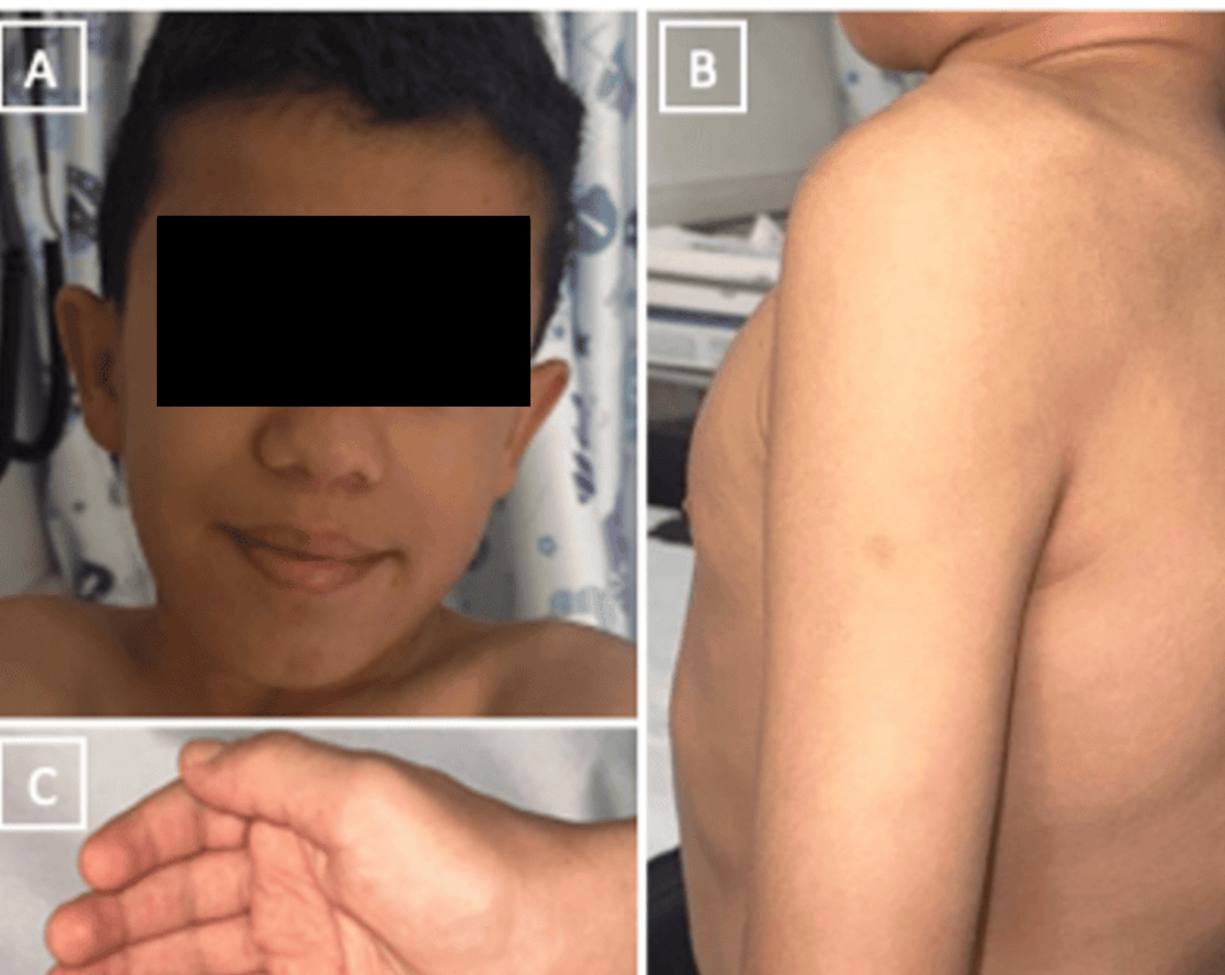
Clinical photograph showing the presenting signs in Patient 3 An 11-year-old child with (A) coarse facial features, (B) thoracolumbar kyphosis, and (C) angiokeratoma of the hand.

At the age of ten, during his visits to the Orthopedics clinic, his short stature was noted, leading to a referral to the Pediatric general clinic. On examination, height drops of two standard deviations, coarse facial features, kyphoscoliosis, and an ejection systolic murmur (grade 2/6) all over the precordium were appreciated (Figure 5A, 5B). Moreover, notable findings included nystagmus poor vision and significant angiokeratomas on both hands, more prominent on the palmar sides (Figure 5C). Based on the presenting symptoms and the findings of the clinical examination, which raised suspicion of GS, multidisciplinary work-ups were initiated, including genetic testing. 

Accordingly, genetic testing was sent to Germany for analysis. Targeted mutation analysis of the CTSA gene using polymerase chain reaction, identified a homozygous mutation (c.607C>A, p.Pro203Thr), confirming the diagnosis of GS at the age of 11. Counseling was given to the family by the metabolic consultant about the risk of recurrence and available options for prenatal genetic diagnosis. 

Further work-up included a growth hormone stimulation test, which yielded a low insulin-like growth factor (34 mcg/L; normal range 64-450 mcg/L), for which the patient is on regular follow-ups with the endocrinologist. The patient was started initially on growth hormone (somatotropin) therapy (30 mcg/kg subcutaneously), resulting in an increase in height of approximately 4.5 cm over a period of six months, with subsequent dosage increases.

During cardiac assessment, the patient reported tiredness during physical activity. An ejection systolic murmur (grade 2/6) at the left sternal border was appreciated. The echocardiography revealed aortic regurgitation, mitral regurgitation, mild left ventricular hypertrophy (LVH), and mild diastolic dysfunction, prompting the initiation of ACE inhibitors. 

**Figure 6:**
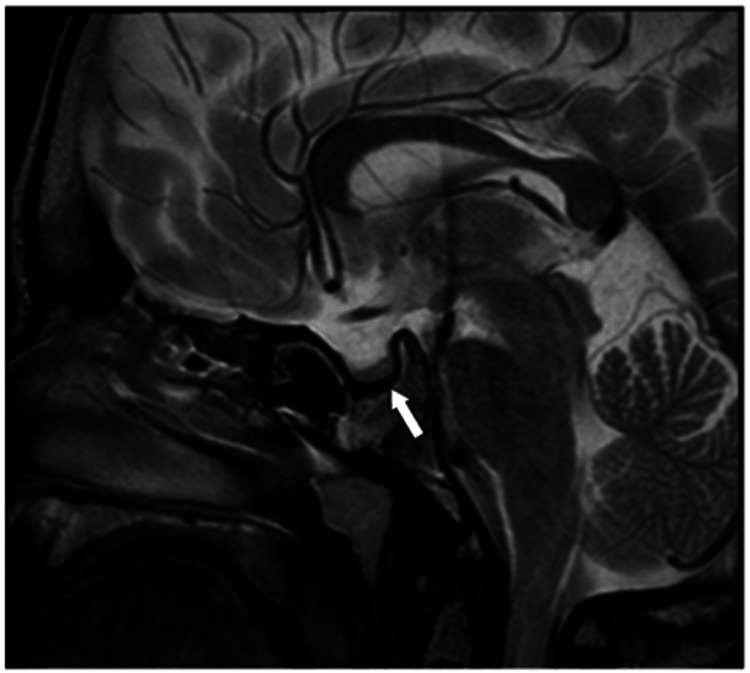
Sagittal T2 MRI of the brain in Patient 3 Enlarged sella turcica and diminished pituitary gland. The imaging correlates with impaired growth and suggests the presence of a partially empty sella turcica. MRI: magnetic resonance imaging

Moreover, an MRI of the brain (Figure 6) showed a greater sella turcica and a small pituitary gland, which correlates with poor growth and indicates a partially empty sella turcica. Neurological evaluation showed normal findings with no focal deficits, no ataxia, and a normal gait except for multidirectional nystagmus, for which the patient is on regular follow-up with Ophthalmology. 

**Figure 7:**
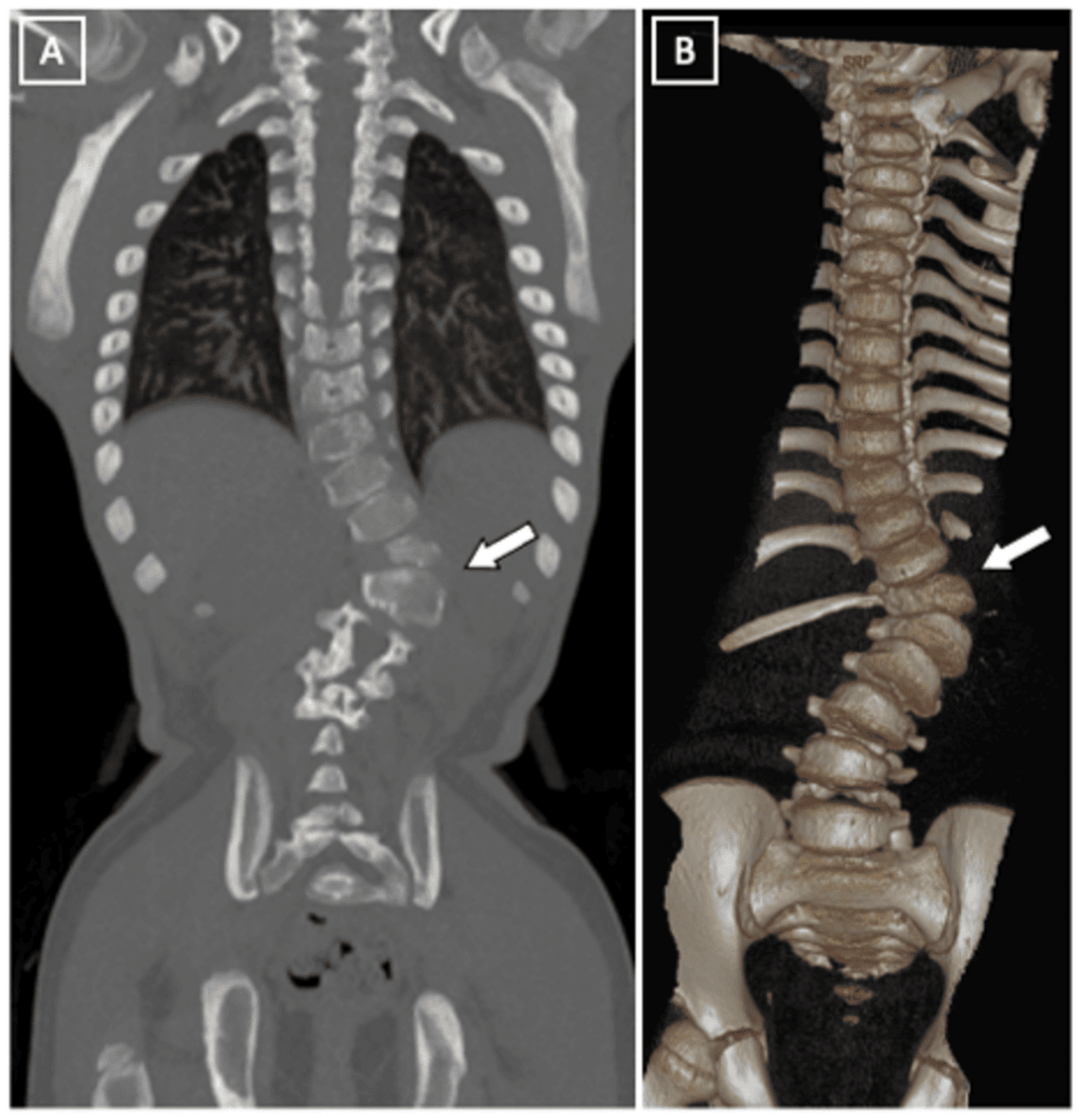
Whole spine CT scan showing a coronal view (A) and a 3D reconstruction (B) The imaging reveals significant thoracolumbar levoscoliosis, with the maximum convexity observed at the T12-L1 level.

Further imaging, including a CT scan of the thoracic spine (Figure 7), was conducted for progressive back pain and showed marked thoracolumbar levoscoliosis, with maximum convexity seen at thoracic spine T12-lumbar spine L1, and no spinal canal stenosis.

The patient was evaluated by a respiratory specialist due to recurrent, prolonged dry cough, increasing in intensity at night. He was diagnosed with bronchial asthma at the age of 11 and is currently on prophylactic budesonide/formoterol. 

Currently, the patient is receiving supportive treatment and is under regular multidisciplinary follow-up with metabolic diseases and genetics, endocrinology, cardiology, orthopedics, and respiratory clinics. 

## Discussion

GS is considered one of the rarest genetic disorders with an autosomal recessive inheritance, first reported by Goldberg et al. [16]. Its exact prevalence remains unclear. Given its rarity, it is often underdiagnosed or misdiagnosed, which can affect prevalence estimates. To date, nearly 157 cases have been reported worldwide, including nine in the Kingdom of Bahrain. Ethnically, 40% of affected individuals have Japanese origins, followed by 4% Portuguese [5,8,14,15].

GS clinically is further classified into three subtypes: EI, late infantile, and juvenile/adult type [5]. In our presented cases, the clinical symptoms and familial patterns are consistent with the late infantile type of GS, with symptoms emerging in the early years of life. The late infantile phenotype is characterized by corneal clouding, cardiac involvement, particularly with thickening of the mitral and aortic valves, and visceromegaly, as well as hearing loss [2,6,8,11]. Seizures and overt neurologic signs can be seen, but their manifestation is rare. Patients with the late infantile form mostly survive into adulthood [6,8,11]. Similarly, all three of our patients showed coarse facial features, short stature, poor vision, along with musculoskeletal pain. Additionally, Patient 3 has large angiokeratomas on both hands. 

Notably, all patients are from consanguineous parents; Patients 1 and 2 are siblings. As the disease is inherited as autosomal recessive, finding more than one patient in the same family is not uncommon. This was also identified in the previous nine cases reported from Bahrain [5].

In Patient 1, echocardiography showed slight mitral stenosis and regurgitation, aortic stenosis, and tricuspid valve stenosis, while in Patient 2, valvular manifestations (thickened mitral valve, mitral valve regurgitation, tricuspid regurgitation, and pulmonary regurgitation) were shown. Patient 3’s echocardiography displayed diastolic dysfunction, mild LVH, aortic regurgitation, and mitral regurgitation. All cardiac manifestations in our patients were previously linked to GS by Annunziata et al. [6]. 

Regarding musculoskeletal manifestations, Patient 1 showed significant bilateral hip osteoarthritis and various skeletal anomalies. Patient 2 showed lumbar lordosis, pectus carinatum, short stature, and coarse facial features. Imaging tests at the age of 12 revealed spondyloepiphyseal dysplasia congenita and signs of early Perthes disease in the left femoral head, ultimately necessitating total hip replacement due to extensive bilateral hip avascular necrosis, while Patient 3 has thoracolumbar levoscoliosis along with back pain and short stature. The musculoskeletal system is commonly affected, leading to different levels of musculoskeletal deformities [2]. Few studies have noted articular manifestations in patients, but understanding of GS-related joint pathology is still limited [1]. A case of late infantile form exhibits a remarkable severity of osteoarticular abnormalities, showing recurrent severe relapsing inflammatory arthritis in both knees, a condition that has not been previously reported [1].

Diagnosis of GS is confirmed through CTSA* *gene mutation testing or enzyme assay methods [17]. Galjart et al. discovered the complementary DNA CTSA gene on chromosome 20q13.1 in 1988 [18]. A total of 35 CTSA gene mutations have been reported in all cases of GS, including small deletions/insertions, missense mutations, splicing variants, and nonsense mutations [19]. Another diagnostic method involves the detection of β-GAL and α-neuraminidase enzyme activities in fibroblasts or leukocytes [19].

In our cases, the identified CTSAgene mutation confirmed the diagnosis. The same homozygous mutation (c.607C>A, p.Pro203Thr) was found through targeted mutation analysis in all three patients. This genetic variation results in the substitution of threonine for proline at amino acid 203, likely being deleterious due to the high conservation of this residue [5]. Nine out of 10 bioinformatics programs predict this variant to be damaging [5]. Moreover, this mutation has been reported in previous research in 2022 in nine Bahraini patients as a novel mutation [5]. Thus, this positive targeted mutation is identified as a founder mutation associated with GS, geographically isolated within the Bahraini population. Founder mutations, or founder variants, are disease-causing genetic changes that occur frequently in geographically or culturally isolated groups whose shared ancestors carried the altered gene [20-22]. Practices of non-random mating, known as endogamy, result in a limited genetic pool and increased genetic homogeneity [20,22]. In Middle Eastern Arab populations, where consanguinity is more common, this effect can be intensified [23,24]. These factors, combined with a high carrier rate of common founder mutations, contribute to a greater prevalence of certain rare genetic diseases compared to the general population and globally [23,24].

Considering all of these, the diagnosis for the three cases was determined using only targeted mutation analysis instead of full Sanger sequencing or whole exome sequencing, making the diagnostic process faster and more cost-effective. 

From a therapeutic point of view, treatments have mainly focused on supportive care and the management of complications. Clinical trials have been reported for two long-term treatment options, including the PPCA-mediated enzyme replacement therapy and adeno-associated virus (AAV)-mediated in vivo gene therapy, both of which have shown promising results in model mice affected by GS [25,26]. 

## Conclusions

GS is a rare autosomal recessive lysosomal storage disease caused by a mutation in the cathepsin CTSA gene, which encodes lysosomal proteins. We report three new cases in Bahrain, all sharing the same homozygous mutation (c.607C>A, p.Pro203Thr). Although these cases were not related to the previously reported patients in Bahrain, the founder mutation makes it easy to diagnose more cases through target mutation. This case series highlights the disease manifestations, particularly skeletal deformities, which significantly affect the patient's quality of life. Moreover, it underscores the need for further research and encourages clinicians to recognize these manifestations to facilitate faster diagnosis and earlier management plans for affected patients. The disease still lacks further studies on the treatment options that can ultimately change the disease outcome. 
